# A study on the application of radiomics based on cardiac MR non-enhanced cine sequence in the early diagnosis of hypertensive heart disease

**DOI:** 10.1186/s12880-024-01301-9

**Published:** 2024-05-27

**Authors:** Ze-Peng Ma, Shi-Wei Wang, Lin-Yan Xue, Xiao-Dan Zhang, Wei Zheng, Yong-Xia Zhao, Shuang-Rui Yuan, Gao-Yang Li, Ya-Nan Yu, Jia-Ning Wang, Tian-Le Zhang

**Affiliations:** 1 Department of Radiology, Affiliated Hospital of Hebei University/ Clinical Medical College, Hebei University, Baoding, 071000 China; 2 Hebei Key Laboratory of Precise Imaging of Inflammation Related Tumors, Baoding, 071000 China; 3 College of Quality and Technical Supervision, Hebei University, Baoding, 071002 China; 4 Department of Ultrasound, Affiliated Hospital of Hebei University, 212 Yuhua East Road, Baoding, 071000 China; 5 College of Electronic and Information Engineering, Hebei University, Baoding, 071002 China

**Keywords:** Hypertensive heart disease, Latent heart damage, Cardiac magnetic resonance, Non-enhanced cine sequence, Radiomics

## Abstract

**Background:**

The prevalence of hypertensive heart disease (HHD) is high and there is currently no easy way to detect early HHD. Explore the application of radiomics using cardiac magnetic resonance (CMR) non-enhanced cine sequences in diagnosing HHD and latent cardiac changes caused by hypertension.

**Methods:**

132 patients who underwent CMR scanning were divided into groups: HHD (42), hypertension with normal cardiac structure and function (HWN) group (46), and normal control (NOR) group (44). Myocardial regions of the end-diastolic (ED) and end-systolic (ES) phases of the CMR short-axis cine sequence images were segmented into regions of interest (ROI). Three feature subsets (ED, ES, and ED combined with ES) were established after radiomic least absolute shrinkage and selection operator feature selection. Nine radiomic models were built using random forest (RF), support vector machine (SVM), and naive Bayes. Model performance was analyzed using receiver operating characteristic curves, and metrics like accuracy, area under the curve (AUC), precision, recall, and specificity.

**Results:**

The feature subsets included first-order, shape, and texture features. SVM of ED combined with ES achieved the highest accuracy (0.833), with a macro-average AUC of 0.941. AUCs for HHD, HWN, and NOR identification were 0.967, 0.876, and 0.963, respectively. Precisions were 0.972, 0.740, and 0.826; recalls were 0.833, 0.804, and 0.863, respectively; and specificities were 0.989, 0.863, and 0.909, respectively.

**Conclusions:**

Radiomics technology using CMR non-enhanced cine sequences can detect early cardiac changes due to hypertension. It holds promise for future use in screening for latent cardiac damage in early HHD.

## Introduction

Hypertension is a health issue affecting various countries, especially those in the developing world [1]. Chronic hypertension can result in irreversible damage to multiple organs, with the heart being a commonly affected organ [2]. Hypertensive heart disease (HHD) encompasses a range of pathological changes induced by hypertension, including left ventricular hypertrophy, systolic and diastolic dysfunction, and interstitial fibrosis [3, 4]. If left untreated, HHD frequently progresses to arrhythmias and heart failure [5]. Timely and appropriate antihypertensive therapy has the potential to decelerate or reverse left ventricular remodeling in HHD, consequently lowering the risk of adverse cardiovascular events [6, 7]. Therefore, early diagnosis of HHD holds significant importance. Cardiac magnetic resonance (CMR) serves as the gold standard for assessing heart structure, function, and myocardial histological characteristics [8, 9]. Currently, diagnosing HHD in the CMR process often relies on identifying a left ventricular wall thickness > 12 mm, coupled with a history of prolonged hypertension and the absence of other conditions causing myocardial thickening and cardiac function abnormalities [10]. Therefore, an HHD diagnosis through CMR implies structural or functional abnormalities. Therefore, identifying latent heart damage in hypertensive patients with negative CMR findings is crucial for the early diagnosis of HHD.

Radiomics can uncover intricate image details, capturing spatial and intensity nuances beyond the scope of a diagnostician’s visual interpretation. In recent years, the application of radiomics technology in cardiovascular diseases has been increasing [11]. Numerous studies have demonstrated that radiomics analysis based on CMR native T1 imaging and extracellular volume maps can effectively identify HHD and hypertrophic cardiomyopathy [10, 12–14]. However, the utilization of the T1 mapping sequence is limited by additional costs and an extended CMR examination time, rendering it less commonly used in clinical practice. Recognizing the potential of radiomics in extracting comprehensive information for images, research focusing on radiomics based on CMR cine sequences is on the rise. Limited studies have indicated that radiomics based on CMR cine sequences can effectively identify myocardial fibrosis and scar [15–19]. Furthermore, it has demonstrated the capability to differentiate myocardial amyloidosis from hypertrophic cardiomyopathy [20]. However, limited research exists on the application of radiomics based on CMR cine sequences in diagnosing latent heart damage resulting from early hypertension.

In this study, diagnostic models using radiomics were developed based on CMR non-enhanced cine sequences to identify HHD and early cardiac changes induced by hypertension. Additionally, this study aimed to investigate the presence of latent cardiac changes in hypertensive patients with negative CMR.

## Materials and methods

### Patient population

A total of 132 patients, comprising 81 males and 51 females, aged 22–78 years, with a mean age of 49.7 ± 11.6 years and a BMI ranging from 17.3 kg/m2 to 37.6 kg/m2 (mean BMI of 26.5 ± 4.2 kg/m2), were prospectively collected during their visits to our hospital for CMR examination between September 2019 and September 2023. Patients were divided into three groups: HHD, hypertension with normal cardiac structure and function (HWN), and normal control (NOR), based on clinical and CMR diagnostic criteria. The inclusion criteria for the HHD group were: (1) a history of hypertension; (2) left ventricular wall thickness > 12 mm and/or left ventricular ejection fraction (LVEF) < 50%; and (3) exclusion of other cardiac diseases causing myocardial hypertrophy or reduced cardiac function (such as aortic stenosis, ischemic heart disease, cardiomyopathy, and heart disease). The HWN group met the following conditions: (1) a history of hypertension and (2) left ventricular wall thickness < 12 mm and LVEF > 50%. Exclusion criteria for both groups were: (1) diabetes mellitus, (2) history of drinking, and (3) coronary artery disease. The NOR group included healthy adult volunteers and clinical patients without cardiac disease, hypertension, hyperglycemia, or hyperlipidemia. Ultimately, the study included 42, 46, and 44 patients in the HHD, HWN, and NOR groups, respectively. This study was approved by the Ethics Committee of affiliate hospital of Hebei university (the ethical code: HDFYLL-KY-2023-145), and all data were collected in the Department of Radiology, Affiliated Hospital of Hebei University. Informed consent was obtained from all patients or their immediate family members before the examination commenced.

### Magnetic resonance imaging acquisition

All patients underwent scanning using a 3.0 T superconducting magnetic resonance scanner (HD750; GE Healthcare, USA). The patient was positioned in a supine manner with an eight-channel array coil on the anterior thorax, and scans were conducted using electrocardiography and respiratory gating techniques. CMR cine images were acquired using a fast imaging with a steady-state acquisition sequence. The scanning parameters were as follows: repetition time, 3.4 ms; Echo, 1.5 ms; flip angle, 45°; excitation count, 1; scanning field of view, 350 cm × 315 cm; layer thickness, 8 mm; and spacing, 2 mm. Each scanning layer comprised 25 dynamic phases.

### Image segmentation and feature extraction

**Fig. 1:**
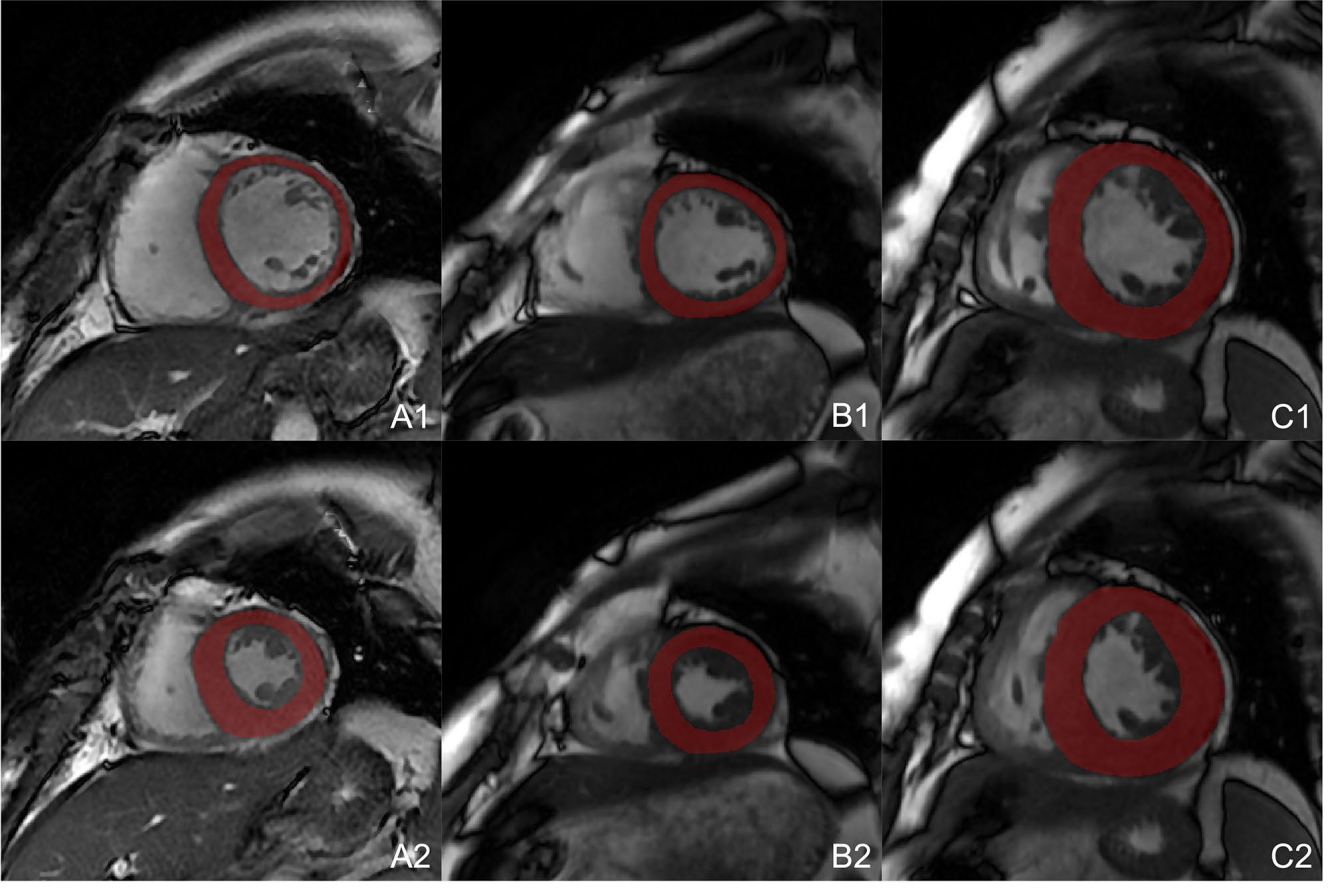
ROI of CMR cine sequences. **A1**, **B1**, and **C1** represent NOR, HWN and HHD during the ED phase, whereas **A2**, **B2**, and **C2** represent NOR, HWN, and HHD of ES phase, respectively

Segmentation of the region of interest (ROI) was performed on the end-diastolic (ED) and end-systolic (ES) phases of CMR left ventricular short-axis cine sequence images using ITK-SNAP software (www.itksnap.org). The selected ROI was the left ventricular myocardium, excluding the trabecula; Fig. 1. Delineation of the ROI was performed manually by an attending physician with > 5 years of CMR experience. All ROI profiles were reviewed by another senior physician with > 10 years of CMR experience for quality control. Both physicians performed the ROI segmentation and review under double-blind conditions.

Feature extraction was performed using the PyRadiomics Toolkit. Various filters, including wavelet transform, Gaussian filter, exponential, gradient, logarithmic, square, and square root, were employed to extract radiomic features, and 1521 features were extracted in both the ED and ES phases. Among these, 111 features were extracted from the original image, 752 features were derived from the wavelet transform, 188 features were obtained through the Laplace operator of the Gaussian filter, and 94 features were extracted respectively from exponent, gradient, logarithm, square, and square roots. Data normalization was achieved using the Z-score, and feature selection was carried out with the least absolute shrinkage and selection operator. Finally, three feature subsets—ED, ES, and ED combined with ES (EDES)—were obtained.

### Classification and validation

Random Forest (RF), support vector machine (SVM), and naive Bayes (NB) machine learning algorithms were employed to construct radiomic diagnostic models. A five-fold cross-validation was performed on all patient data, where all data were evenly divided into five parts. In each experiment, one part was designated as the test set, and the remaining parts were utilized as the training set. Receiver operating characteristic (ROC) curves were employed to assess the predictive efficiency of the models, and various metrics such as the area under the curve (AUC), accuracy, precision, recall, and specificity were calculated.

### Statistical analysis

The statistical analysis of all data was performed using Python (version 3.7) and IBM SPSS (version 26.0). For measurement data with a normal distribution, the results are expressed as the mean ± standard deviation. Data that are not fitting a normal distribution were described using the median (upper and lower quartiles). Count data were expressed as percentages. Analysis of variance was used for continuous data conforming to a normal distribution and homogeneity of variance; otherwise, nonparametric tests were used. The chi-square test was employed to compare categorical variables between groups. The performance of different radiomic models in distinguishing the three groups was evaluated using the ROC curve and accuracy. A significance level of *P* < 0.05 was considered a statistically significant difference.

## Results

### Characteristics of the study population and parameters based on CMR measurements

General patient information and CMR measurement results are displayed in Table 1. No statistically significant differences were observed in age, sex and left ventricular strake volume index (LVSVI) among the three groups (*P* > 0.05). However, significant differences were noted in heart rate, LVEF, left ventricular end-diastolic volume index (LVEDVI), left ventricular end-systolic volume index (LVESVI), and left ventricular sidewall thickness between the HHD group and the HWN and NOR groups (*P* < 0.05). No significant differences were found between the HWN and NOR groups (*P* > 0.05). Furthermore, significant differences in BMI, left ventricular mass index (LVMI), and interventricular septal thickness were observed among the three groups (*P* < 0.001).

**Table 1:** study population and CMR characteristics

	HHD (*n* = 42)	HWN (*n* = 46)	NOR (*n* = 44)	*P* value
Age (years)	49.6 ± 14.3	52.1 ± 8.5	47.3 ± 11.2	0.15
Male/Female	27/15	29/17	19/25	0.08
BMI (kg/m^2^)	28.9 ± 4.5	26.6 ± 3.3	24.0 ± 3.3	< 0.001^a b c^
Heart rate (beats/min)	74.9 ± 13.1	69.7 ± 11.1	68.0 ± 11.9	0.04^a c^
LVEF (%)	42.3 ± 20.7	66.3 ± 7.0	66.1 ± 5.7	< 0.001^a c^
LVEDVI (ml/m^2^)	120.3 ± 48.2	74.6 ± 12.8	75.6 ± 13.2	< 0.001^a c^
LVESVI (ml/m^2^)	75.8 ± 46.9	25.1 ± 6.9	25.9 ± 7.7	< 0.001^a c^
LVSVI (ml/m^2^)	44.4 ± 18.9	49.5 ± 9.6	49.7 ± 8.0	0.25
LVMI (g/m^2^)	106.6 ± 31.5	63.6 ± 12.1	54.1 ± 8.4	< 0.001^a b c^
LVWT^1^ (mm)	13.3 ± 3.7	10.2 ± 1.8	8.0 ± 1.6	< 0.001^a b c^
LVWT^2^ (mm)	10.1 ± 2.8	7.2 ± 1.2	6.5 ± 1.4	< 0.001^a c^

HHD, hypertensive heart disease; HWN, Hypertension with normal cardiac structure and function; NOR, normal; LVEF, left ventricular ejection fraction; LVEDVI, left ventricular end-diastolic volume index; LVESVI, left ventricular end-systolic volume index; LVSVI, left ventricular strake volume index; LVMI, left ventricular mass index; LVWT^1^, left ventricular wall thickness of interventricular septum; LVWT^2^, left ventricular sidewall thickness. Values are given as mean ± standard deviation for continuous variables; and count (%) for categorical variables. ^a^ HHD vs. NOR; ^b^ HWN vs. NOR; ^c^ HHD vs. HWN

### Radiomics features selection

**Fig. 2:**
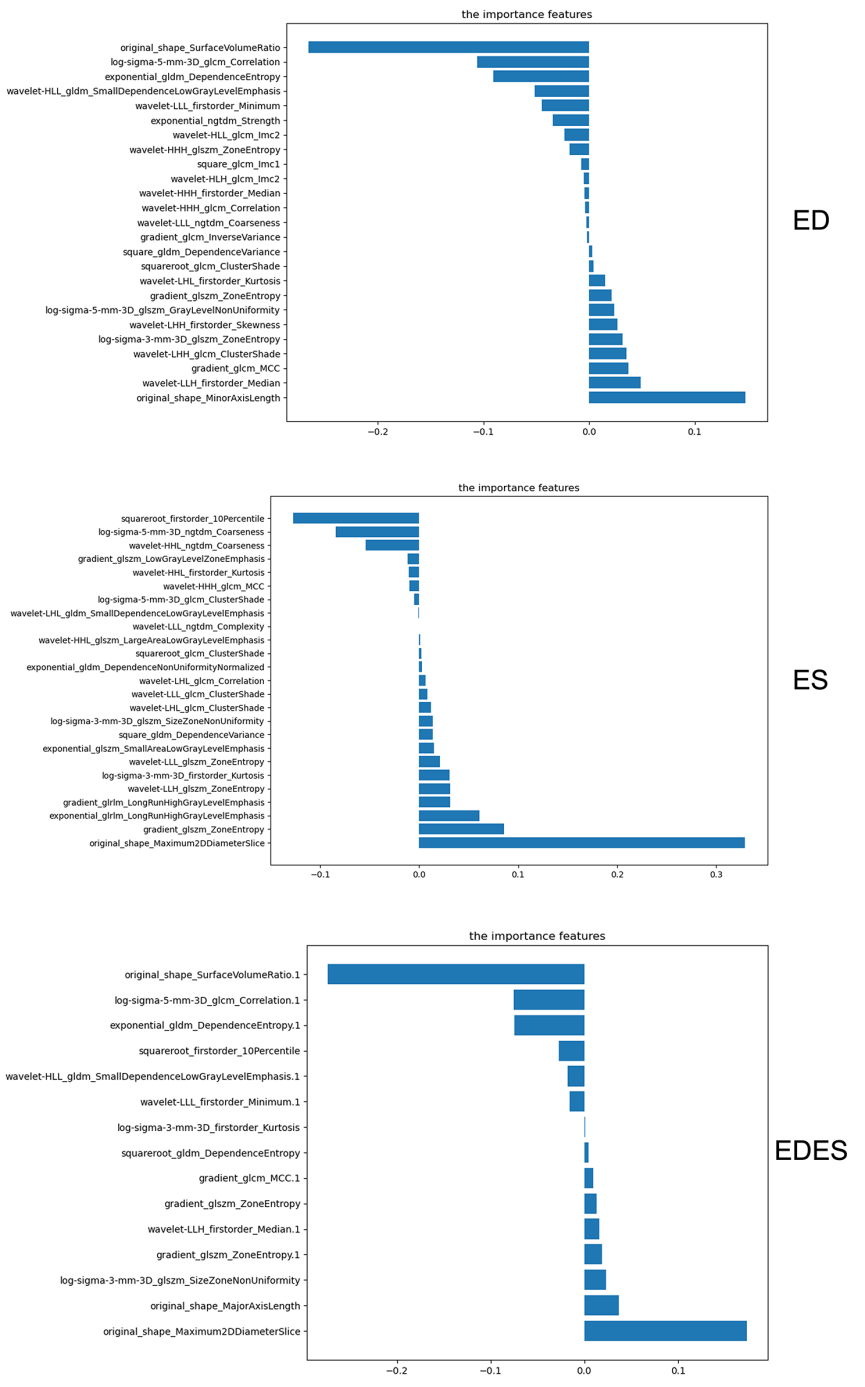
Histogram of feature coefficients of different feature subsets. The features in EDES whose names end in “.1” are extracted from ED and the rest from ES. (The features extracted from ED in EDES are: original_shape_SurfaceVolumeRatio, log-sigma-5-mm-3D_glcm_Correlation, exponential_gldm_DependenceEntropy, wavelet-HLL_gldm_SmallDependenceLowGrayLevelEmphasis, wavelet-LLL_firstorder_Minimum, gradient_glcm_MCC, wavelet-LLH_firstorder_Median, and gradient_glszm_ZoneEntropy; The features extracted from ES are: squareroot_firstorder_10Percentile, log-sigma-3-mm-3D_firstorder_Kurtosis, squareroot_gldm_DependenceEntropy, gradient_glszm_ZoneEntropy, log-sigma-3-mm-3D_glszm_SizeZoneNonUniformity, original_shape_MajorAxisLength, and original_shape_Maximum2DDiameterSlice.) The most important features include surface area to volume ratio from ED and maximum 2D diameter (slice) from ES

The results of the radiomic feature selection for ED, ES, and EDES are depicted in Fig. 2. The results showed that the selected radiomic features from the original image had the most significant impact on classification. However, the results from other filters also positively contributed to the classification. Specifically, 25 features were selected from the ED, including five first-order features, two shape features, and 18 texture features. The feature with the highest importance was original_shape_SurfaceVolumeRatio. For ES, 25 features were selected, including three first-order features, one shape feature, and 21 texture features, with the feature of highest importance being original_shape_Maximum2DDiameterSlice. In the case of EDES, 15 features were selected, encompassing four first-order features, three shape features, and eight texture features, with the feature of highest importance being ED_original_shape_SurfaceVolumeRatio.

### Radiomics model construction and diagnostic performance evaluation

**Fig. 3:**
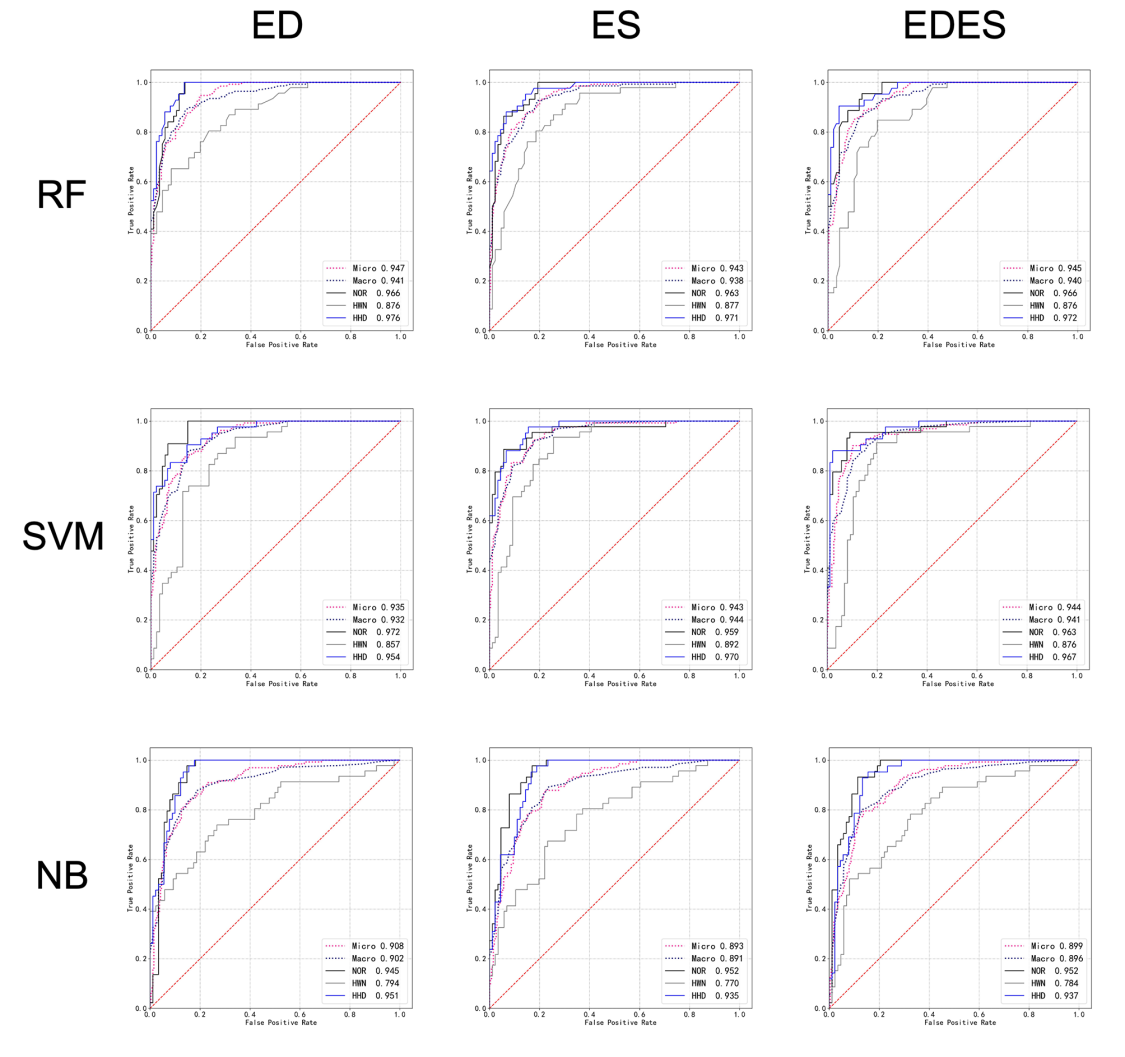
ROC curves of different radiomic models

The feature subsets of ED, ES, and EDES were trained using RF, SVM, and NB machine learning algorithms, resulting in nine radiomic diagnostic models to differentiate between HHD, HWN, and NOR. The prediction results for the nine models are listed in Table 2. The ROC curves for each model are illustrated in Fig. 3. Notably, the RF model of ED and the RF and SVM models of ES and EDES demonstrated high macro-averaged AUC values of 0.94. Specifically, the SVM model of EDES achieved the highest accuracy of 0.833, surpassing the other models by 1–9% points. Its AUC values for predicting HHD, HWN, and NOR were 0.967, 0.876, and 0.963, with precisions of 0.972, 0.740, and 0.826; recalls of 0.833, 0.804, and 0.863; and specificities of 0.989, 0.863, and 0.909, respectively.

**Table 2:** ROC curve analysis results of different radiomacs diagnostic models

	Classifier	Accuracy	AUC			macro-average AUC	Precision/ Recall/ Specificity		
			HHD	HWN	NOR		HHD	HWN	NOR
ED	RF	0.788	0.976	0.876	0.966	0.941	0.833/ 0.833/ 0.922	0.841/ 0.674/ 0.849	0.826/ 0.864/ 0.909
	SVM	0.787	0.954	0.857	0.972	0.932	0.846/ 0.785/ 0.933	0.680/ 0.739/ 0.863	0.860/ 0.840/ 0.932
	NB	0.765	0.951	0.794	0.945	0.902	0.776/ 0.905/ 0.878	0.759/ 0.478/ 0.919	0.759/ 0.932/ 0.824
ES	RF	0.812	0.971	0.877	0.963	0.938	0.895/ 0.810/ 0.955	0.714/ 0.761/ 0.837	0.844/ 0.863/ 0.920
	SVM	0.795	0.970	0.892	0.959	0.944	0.804/ 0.881/ 0.900	0.727/ 0.696/ 0.860	0.857/ 0.818/ 0.932
	NB	0.742	0.953	0.770	0.952	0.891	0.727/ 0.952/ 0.833	0.773/ 0.370/ 0.942	0.745/ 0.932/ 0.841
EDES	RF	0.826	0.972	0.876	0.966	0.940	0.923/ 0.857/ 0.967	0.756/ 0.739/ 0.872	0.813/ 0.867/ 0.898
	SVM	0.833	0.967	0.876	0.963	0.941	0.972/ 0.833/ 0.989	0.740/ 0.804/ 0.863	0.826/ 0.863/ 0.909
	NB	0.765	0.937	0.784	0.952	0.896	0.736/ 0.929/ 0.844	0.778/ 0.457/ 0.932	0.788/ 0.932/ 0.875

## Discussion

Hypertension stands as a significant risk factor for human health, with its global incidence witnessing an annual increase. HHD is a common condition resulting from hypertension, posing a heightened risk of adverse cardiovascular events and significantly impacting quality of life. Numerous studies indicate a rising prevalence and mortality associated with HHD over the past three decades, with projections anticipating a continued increase in HHD mortality in the next decade [21–23]. Therefore, early diagnosis and intervention of HHD are paramount concerns for clinicians. As a comprehensive cardiac examination method, CMR offers several advantages in diagnosing HHD. Notably, the CMR cine sequence eliminates the need for gadolinium contrast agents. The resulting images provide both structural and functional information about the heart, and the subtle differences in signal characteristics within the images may escape visual recognition by CMR physicians. Therefore, the full potential of this sequence is currently not fully explored. Radiomics technology has become a prominent research topic in recent years, due to its ability to identify image differences that may not be visually recognized by CMR physicians. This technology delves into subtle differences and lesions within the images, thereby enhancing the detection of early changes in cardiac structure and tissue [24].

The results of this study revealed a significant difference in BMI among individuals HHD, HWN, and NOR (28.9 ± 4.5 vs. 26.6 ± 3.3 vs. 24.0 ± 3.3). This finding aligns with those of previous studies showing that hypertension and HHD are associated with a higher body weight or obesity [25, 26]. Our study’s results imply that a higher BMI correlates with an increased likelihood of causing heart damage, potentially leading to HHD. Furthermore, this study observed that the thicknesses of the interventricular septum and left ventricular sidewall in patients with HHD were greater than those in individuals with hypertension and the healthy population (*P* < 0.001). Although the interventricular septum thickness did not exceed the normal ventricular wall thickness (12 mm) in patients with hypertension, it was notably higher than that in the healthy population (10.2 ± 1.8 vs. 8.0 ± 1.6, *P* < 0.05). No statistically significant difference was observed in the left ventricular sidewall thickness between the two groups. Therefore, these findings suggest that myocardial thickening induced by hypertension may initiate in the interventricular septum. Moreover, the results imply that the diagnostic criteria for ventricular wall thickening in patients with hypertension should be more stringent.

Currently, limited research exists on radiomics for the early diagnosis of HHD, with only one radiomics study by Cetin et al. focusing on early cardiac changes caused by hypertension being identified [27]. Cetin et al. extracted radiomic features from the myocardial ring as well as the left and right ventricles in their study. However, in our study, radiomics features were solely extracted from the myocardial ring. This decision was made considering that a substantial signal variation in the ventricular blood pool might impact the study results. Additionally, the endomyocardial ring was deemed sufficient for reflecting the left ventricular morphology and size. In this study, features from ED, ES, and EDES phases were selected separately. All three feature subsets comprised first-order, shape, and texture features. The most crucial features across all feature subsets were shape features: the surface-area-to-volume ratio in the ED phase and the maximum 2D diameter (slice) in the ES phase. This indicates that a larger myocardial volume under the same surface area and greater myocardial diameter in the axial plane of the heart are associated with an increased likelihood of developing HHD. These findings align with our understanding that cardiac changes induced by hypertension often manifest as increased myocardial volume and heart enlargement. Among the three feature subsets, texture features constituted the largest proportion accounting for 72% in ED, 84% in ES, and 53% in EDES. This suggests that myocardial texture features play a crucial role in identifying HHD, suggesting that hypertension may contribute to the heterogeneity of tissue texture in the myocardial microstructure. In contrast to the findings of Cetin et al., this study revealed that both shape and texture features were of significant important. Cetin et al. had previously concluded that early heart changes caused by hypertension did not involve shape or size. The discrepancy in findings may be attributed to several factors. Firstly, this study employed a triple classification, including data from HHD that were not part of Cetin et al.‘s study. Notably, HHD exhibits obvious morphological changes in the heart compared with a normal heart. Second, differences in the quantity of data and the delineation of the ROI between the two studies could also contribute to inconsistencies in identifying important features.

The ROC curve analysis of various models demonstrated that the radiomic model exhibited higher efficiency in identifying healthy heart and HHD, with an AUC > 0.96. It displayed slightly lower efficiency in identifying hypertension without cardiac abnormalities, although the AUC still reached 0.876. The combination of feature from ED and ES phases improved accuracy, with SVM classifier proving to be the most effective. The diagnostic model established in this study showed improved classification of patients with HHD, hypertensive patients, and healthy individuals. This suggests the possibility of latent cardiac changes in hypertensive patients with a normal CMR diagnosis, detectable and diagnosable using radiomic technology based on CMR cine sequences.

Since the amount of data in this study is not very large, we adopted the method of manual annotation. Manual annotation can be used as the gold standard for CMR image segmentation. Subsequent studies will continue to increase the amount of data for training and validation, so the workload of manual annotation will increase significantly. In addition, manual segmentation is prone to produce inter-observer and intra-observer variability, which is not conducive to the reproducibility of radiomics model. Using semi-automatic or automatic segmentation may be the best solution. There have been a number of studies on segmentation algorithms have achieved good segmentation results [28, 29]. However, CMR images are susceptible to noise and artefacts [30], which can reduce the accuracy of automatic segmentation and radiomics analysis. Stochastic resonance can be used to improve the contrast by using the noise in the image, so that more accurate segmentation can be performed [31, 32]. At present, studies have shown that stochastic resonance can enhance the recognition of myocardial contours in CMR images and improve the accuracy of segmentation [33, 34]. In addition, deep learning (DL) technology is also widely used for segmentation and classification of CMR images [35]. The application of DL is more conducive to the automation of the entire diagnostic process [36], and it can improve the accuracy and repeatability of model diagnosis while improving the efficiency of CMR image segmentation [37]. One study showed that DL performed better than traditional machine learning techniques when classifying CMR images with complex cardiac anatomy [38]. Therefore, we believe that the future stochastic resonance and DL will help improve the accuracy, repeatability and automation of radiomics models for early HHD diagnosis.

This study has several limitations. First, it only extracted features from myocardial ring and did not analyze the papillary muscles and left atrium, potentially resulting in the loss of some image information. Second, being a single-center study with a small sample size, external validation was not conducted. In the future, we will continue to expand the sample to train the model using multi-centre data and perform external validation to enhance the robustness and repeatability of the model. In addition, the feature selection process in this study used all the data, therefore the models were exposed to all the data in some capacity during the classification stage, which may lead to an overestimation of performance. Next, we will continue to expand the sample size and only use the data of the training set for feature selection, so as to obtain more reliable results and improve the robustness of the model. Finally, factors such as illness duration and hypertension grade were not considered in this study. Future research will involve ongoing follow-ups of hypertensive patients to explore which radiomics features are associated with the progression of HHD to cardiac abnormalities.

## Conclusion

Radiomics technique based on CMR non-enhanced cine sequences proves effective in identifying patients with HHD, individuals with hypertension, and those who are healthy. Notably, patients with hypertension who receive a normal CMR diagnosis may still harbor latent cardiac changes, detectable through radiomics technique.

## Data Availability

The datasets used during the current study are available from the corresponding author upon reasonable request.

## Abbreviations

HHD: Hypertensive heart disease
CMR: Cardiac magnetic resonance
HWN: Hypertension with normal cardiac structure and function
NOR: Normal control
LVEF: Left ventricular ejection fraction
ROI: Region of interest
ED: End-diastolic
ES: End-systolic
EDES: End-diastolic combined with End-systolic
RF: Random forest
SVM: Support vector machine
NB: Naive bayes
ROC: Receiver operating characteristic
AUC: Area under the curve
LVSVI: Left ventricular strake volume index
LVEDVI: Left ventricular end-diastolic volume index
LVESVI: Left ventricular end-systolic volume index
LVMI: Left ventricular mass index
DL: Deep learning
